# Genetic polymorphisms in cyclin D1 are associated with risk of renal cell cancer in the Chinese population

**DOI:** 10.18632/oncotarget.20720

**Published:** 2017-09-08

**Authors:** Jianxin Xue, Zhiqiang Qin, Xiao Li, Jianzhong Zhang, Yuxiao Zheng, Weizhang Xu, Qiang Cao, Zengjun Wang

**Affiliations:** ^1^ Department of Urology, The First Affiliated Hospital of Nanjing Medical University, Nanjing, 210029, China; ^2^ Department of Urology, The Second Affiliated Hospital of Southeast University, Nanjing, 210003, China; ^3^ Department of Urologic Surgery, The Affiliated Cancer Hospital of Jiangsu Province of Nanjing Medical University, Nanjing, 210009, China; ^4^ Department of Thoracic Surgery, Nanjing Medical University Affiliated Cancer Hospital, Jiangsu Key Laboratory of Molecular and Translational Cancer Research, Cancer Institute of Jiangsu Province, Nanjing, 210009, China

**Keywords:** cyclin D1, polymorphism, renal cell cancer, genetic susceptibility

## Abstract

Recently, the functional polymorphisms in Cyclin D1 (CCND1) have been shown the potential influence to risk of renal cell cancer (RCC). Therefore, the present study was performed to investigate whether these polymorphisms could influence the susceptibility of RCC. Four potentially functional polymorphisms in CCND1 (rs1944129, rs7177, rs9344 and rs678653) were genotyped in this hospital-based case-control study, comprising of 1,488 RCC patients and 1,677 cancer-free controls in a Chinese population by the TaqMan assay. The logistic regression was used to assess the associations between CCND1 polymorphisms and the risk of RCC. We found the genotype and allele frequency distribution of rs1944129 and rs7177 were significantly associated with risk of RCC (*P* = 0.015 and *P* = 0.018, respectively). The analysis of combined risk alleles revealed that patients with 2-4 risk alleles showed an elevated risk of RCC compared to those with 0-1 risk alleles (OR = 1.35, 95% CI = 1.15 - 1.58, *P* < 0.001). Furthermore, compared with the genotypes containing G allele (AG and GG), the patients carrying the AA genotype in CCND1 rs1944129 polymorphism had a significantly greater prevalence of high clinical stage disease (OR = 0.56, 95% CI = 0.33 - 0.94, *P* = 0.029). These results suggested that these CCND1 polymorphisms rs1944129 and rs7177 might contribute to the susceptibility of RCC in the Chinese population.

## INTRODUCTION

As a complex-trait disease, renal cell cancer (RCC) is considered as one of the most common type of the kidney malignancy with a high prevalence in older men (>70 years of age) and is estimated to account for approximately 90% of all renal malignancies [1–4]. Besides, the incidence of RCC is steadily increasing and indicates variation among different populations [4]. The accurate etiology of RCC is not completely well-understood, although its development is involved in a series of complex environmental and lifestyle factors, including obesity, ethnicity, age, tobacco smoking, alcohol consumption, diabetes, hypertension, occupational exposures to chemicals and family history [3, 5–8]. However, recent studies have demonstrated that genetic variations, particularly single-nucleotide genetic polymorphisms (SNPs) might contribute greatly to the occurrence of RCC [9].

As a member of the D-type cyclin family, cyclin D1 (CCND1) was a proto-oncogene and a good biomarker for tumor progression, found to be deregulated in several cancers, including RCC [10]. CCND1 gene, located on chromosome 11q13, is associated with cell proliferation and differentiation, which fulfills a specific role during the cell cycle progression, subsequently affecting the transcription of genes and promoting the growth (G1) phase to the synthesis (S) phase transition of the cell cycle by activating and adhering to cyclin-dependent kinase 4 (CDK4) or cyclin-dependent kinase 6 (CDK6) [11, 12]. CCND1 encodes a subunit of the holoenzyme complex that phosphorylates the retinoblastoma protein (Rb) by CDK4 and CDK6. Phosphorylation of Rb releases transcription factors such as E2F to activate the transcription of genes, whose products are required to entry into S phase of the cell cycle [13, 14]. In addition, the accumulating evidences discoveried that increased expression of CCND1 disrupted normal cell cycle process and possibly promoted the development of many malignant cancers, including RCC, which might be closely associated with metastases and the poor prognosis [15–17].

Previous genome-wide association studies (GWAS) have identified many risk loci for RCC, and expression quantitative trait analyses suggested plausible candidate genes at these regions that might contribute to RCC susceptibility [18]. However, some genetic polymorphisms associated with the risk of RCC still did not be found. Considering that CCND1 played an important role in the occurrence of RCC, we hypothesized that the polymorphisms of this gene might act as a potential genetic marker to predict RCC risk and progression. To validate this hypothesis, we genotyped these polymorphisms in a case-control study, including 1,488 cases and 1,677 controls in a Chinese population in the present study. Therefore, SNPs in CCND1 (rs1944129, rs7177, rs9344 and rs678653) were selected and their genetic associations with RCC risk were evaluated to identify whether genetic variants in CCND1 might contribute to the susceptibility of RCC.

## RESULTS

### Characteristics of RCC patients and controls

The frequency distributions of selected characteristics of the 1,488 cases and 1,677 controls were presented in Table 1. No significant differences between the cases and controls with regard to age, sex and family history of cancer was found (all *P* > 0.05). Nevertheless, there were more subjects with BMI, smokers, drinking status, hypertension patients and diabetics among the cases than among the control subjects (*P* = 0.008, 0.008, 0.036, <0.001 and <0.001, respectively). Of 1,488 patients, 64.0% of the patients were in stage I, whereas 20.0%, 8.0% and 8.0% were found to have stage II, III and IV diseases, respectively. The percent of nuclear grade from I to IV was 21.0%, 50.0%, 22.0% and 7.0% respectively. Moreover, the majority of patients (84.0%) had the conventional RCC. These variables were further adjusted with multivariate logistic regression models.

**Table 1:** Distribution of selected variables between the renal cell cancer cases and the control subjects

Variables	Cases (n = 1488)		Controls (n = 1677)		P -value*
	N	%	N	%	
Age (mean ± SD), years	56.7±12.0		57.8±12.0		0.176
<60	893	60.01	975	58.14	0.285
≥60	595	39.99	702	41.86	
Sex					
Male	942	63.31	1113	66.37	0.072
Female	546	36.69	564	33.63	
BMI (mean ± SD), kg/m^2^	24.1±2.9		23.9±3.1		0.013
<24	730	49.06	902	53.79	0.008
≥24	758	50.94	775	46.21	
Smoking status					
Never	922	61.96	1115	66.49	0.008
Ever	566	38.04	562	33.51	
Drinking status					
Never	1092	73.39	1285	76.62	0.036
Ever	396	26.61	392	23.38	
Family					
No	1380	92.74	1535	91.53	0.208
Yes	108	7.26	142	8.47	
Hypertension					
No	907	60.95	1259	75.07	<0.001
Yes	581	39.05	418	24.93	
Diabetes					
No	1295	87.03	1572	93.74	<0.001
Yes	193	12.97	105	6.26	
Clinical stage					
I	946	0.64			
II	303	0.20			
III	115	0.08			
IV	124	0.08			
Grade					
I	319	0.21			
II	744	0.50			
III	322	0.22			
IV	103	0.07			
Histology					
Clear cell	1250	0.84			
Papillary	53	0.04			
Chromophobe	79	0.05			
Unclassified	106	0.07			

* Student's t-test for age and BMI distributions between cases and controls; two sided χ^2^ test for other selected variables between cases and controls.

### Association between RCC risk and genetic polymorphisms of CCND1

Genotype and allele distributions of the remaining four tSNPs in the patients and controls were detailed in Table 2. All genotype frequencies in controls and patients both conformed to HWE. No significant differences in genotype and allele distributions of rs9344 and rs678653 polymorphisms were observed between the cases and controls (*P* = 0.247 and *P* = 0.120, respectively). However, the genotype frequencies for rs1944129 and rs7177 polymorphisms between the cases and controls were significantly different (*P* = 0.015 and *P* = 0.018, respectively). Based on logistic regression analysis, when using the rs1944129 AA genotype as the reference, the GG and AG+CC genotype of the SNP rs1944129 were associated with a significantly increased risk of RCC (GG vs AA: adjusted OR = 1.56, 95% CI = 1.15 - 2.13, *P* = 0.005; AG+CC vs AA: adjusted OR = 1.17, 95% CI = 1.01 - 1.35, *P* = 0.032). In addition, individual with CC and AC+CC genotype in rs7177 had a significantly increased susceptibility to RCC occurrence (CC vs AA: adjusted OR = 1.83, 95% CI = 1.08 - 3.10, *P* = 0.024; AC+CC vs AA: adjusted OR = 1.17, 95% CI = 1.00 - 1.37, *P* = 0.045), compared with individuals carrying rs7177 AA genotype. Similarly, a significantly increased risk of RCC was found in the genotype rs9344 GG compared with the AA genotype (adjusted OR = 1.24, 95% CI = 1.00 - 1.52, *P* = 0.046).

**Table 2:** The basic information of the genotyped polymorphisms in four SNPs in the CCND1 associated with the RCC risk

Polymorphisms	Cases (n = 1488)		Controls (n = 1677)		P*	Adjusted OR (95% CI)*
	N	%	N	%		
rs1944129						
AA	824	55.4	992	59.2	**0.015**	1.00 (reference)
AG	557	37.4	600	35.8	0.145	1.12(0.96-1.30)
GG	107	7.2	85	5.1	**0.005**	**1.56(1.15-2.13)**
AG+GG	664	44.6	685	40.8	**0.032**	**1.17(1.01-1.35)**
A allele	2205	74.1	2584	77.0	**0.006**	
G allele	771	25.9	770	23.0		
rs7177						
AA	1025	68.9	1205	71.9	**0.018**	1.00 (reference)
AC	423	28.4	448	26.7	0.117	1.14(0.97-1.33)
CC	40	2.7	24	1.4	**0.024**	**1.83(1.08-3.10)**
AC+CC	463	31.1	472	28.1	**0.045**	**1.17(1.00-1.37)**
A allele	2473	83.1	2858	85.2	**0.021**	
C allele	503	16.9	496	14.8		
rs9344						
AA	429	28.8	520	31.0	0.247	1.00 (reference)
AG	740	49.7	831	49.6	0.203	1.11(0.94-1.32)
GG	319	21.4	326	19.4	**0.046**	**1.24(1.00-1.52)**
AG+GG	1059	71.2	1157	69.0	0.098	1.17(0.98-1.34)
A allele	1598	53.7	1871	55.8	0.096	
G allele	1378	46.3	1483	44.2		
rs678653						
CC	1079	72.5	1259	75.1	0.120	1.00 (reference)
CG	380	25.5	397	23.7	0.283	1.10(0.93-1.29)
GG	29	1.9	21	1.3	0.199	1.47(0.82-2.65)
CG+GG	409	27.5	418	24.9	0.192	1.11(0.95-1.31)
C allele	2538	85.3	2915	86.9	0.061	
G allele	438	14.7	439	13.1		

* Adjusted for age, sex, BMI, smoking status, drinking status, diabetes and hypertension in logistic regression model. CI, confidence interval; OR, odds ratio.

### Combined analysis between CCND1 polymorphisms and RCC susceptibility

Because two polymorphisms (rs1944129 and rs7177) appeared to be associated with an increased risk of RCC, we combined two SNPs based on the number of the risk alleles, and evaluated the potential interactions of the polymorphisms on the risk of RCC. As listed in Table 3, statistical significance was observed in the combined analysis of risk alleles. Furthermore, we classified the risk alleles into two groups according to the number of risk alleles. We found that the risk of RCC was significantly increased in subjects that carried 2-4 risk alleles compared to those carrying 0-1 risk alleles (adjusted OR = 1.35, 95%CI = 1.15 - 1.58, *P* < 0.001). However, As shown in Table 4, there was no correlation between the combined genotypes in the clinical stage and grade. We found that the frequencies of patients with 2-4 risk alleles were observed no signifiant difference in both the advanced stage of RCC (31.0%) and the localized stage (31.0%) (adjusted OR = 0.98, 95% CI = 0.69 - 1.39, *P* = 0.897). Moreover, no significant difference was detected in the association between 2-4 risk alleles and the patients with well-differentiated RCC (adjusted OR = 1.18, 95%CI = 0.89 - 1.57, *P* = 0.257).

**Table 3:** Analysis between combined risk alleles and RCC Susceptibility

rs1944129 and rs7177	Cases (n = 1488)		Controls (n = 1677)		P*	Adjusted OR (95% CI)*
	N	%	N	%		
Number of risk alleles						
0	768	51.6	893	53.2		1.00 (reference)
1	265	17.8	370	22.1	0.127	0.86(0.71-1.04)
2	375	25.2	358	21.3	**0.018**	**1.24(1.04-1.48)**
3	61	4.1	44	2.6	**0.009**	**1.72(1.15-2.58)**
4	19	1.3	12	0.7	0.280	1.52(0.71-3.24)
Recombined groups						
0–1	1033	69.4	1263	75.3		1.00 (reference)
2–4	455	30.6	414	24.7	**<0.001**	**1.35(1.15-1.58)**

*Two-sided x2 test for either genotype distributions or allele frequencies between the cases and controls. Adjusted for age, smoking status, drinking status and family history of cancer in logistic regression model; 95% CI: 95% confidence interval. The 0–4 represents the numbers of risk alleles within the combined genotypes; the risk alleles used for the calculation were the rs1944129 and rs7177 alleles.

**Table 4:** Association between the combined genotypes of rs1944129 and rs7177 polymorphisms and clinicopathologic characteristics of renal cell carcinoma

Variables	Risk allele				P*	Adjusted OR (95% CI)*
	0-1		2-4			
	N	%	N	%		
Clinical stage						
I + II	867	69	382	31		1.00 (reference)
III + IV	166	69	73	31	0.897	0.98 (0.69-1.39)
Grade						
I + II	745	70	318	30		1.00 (reference)
III + IV	288	68	137	32	0.257	1.18 (0.89-1.57)

* Adjusted for age, sex, BMI, smoking status, drinking status, diabetes and hypertension in logistic regression model. CI, confidence interval; OR, odds ratio.

### Stratified analysis of the two polymorphisms and clinicopathologic characteristics and risk of RCC

After that, stratification analysis of the two polymorphisms (rs1944129 and rs7177) indicated that the increased risk was more pronounced among younger subjects (adjusted OR = 1.42, 95%CI = 1.15 - 1.75, *P* = 0.001), nonsmokers (adjusted OR = 1.44, 95% CI = 1.18 - 1.76, *P* < 0.001), nondrinkers (adjusted OR = 1.36, 95% CI = 1.13 - 1.63, *P* = 0.001), patients without a family history of RCC (adjusted OR = 1.34, 95% CI = 1.13 - 1.58, *P* = 0.001), and nondiabetes (adjusted OR = 1.42, 95% CI = 1.20 - 1.67, *P* < 0.001) (Table 5).

**Table 5:** Association between the combined genotypes of rs1944129 and rs7177 polymorphisms and clear cell renal cell carcinoma in stratified analysis

Variables	Risk allele				P*	Adjusted OR (95% CI)*
	0-1		2-4			
	Case(n,%)	Control(n,%)	Case(n,%)	Control(n,%)		
Age						
<60	616 (59.6)	741 (58.7)	277 (60.9)	234 (56.5)	**0.001**	**1.42 (1.15-1.75)**
≥60	417 (40.4)	522 (41.3)	178 (39.1)	180 (43.5)	0.064	1.27 (0.98-1.64)
Sex						
Male	650 (65.9)	832 (65.9)	292 (64.2)	281 (67.9)	**0.014**	**1.42 (1.07-1.86)**
Female	383 (37.1)	431 (34.1)	163 (35.8)	133 (32.1)	**0.008**	**1.31 (1.07-1.61)**
BMI						
<24	516 (50.0)	683 (54.1)	214 (47.0)	219 (52.9)	**0.029**	**1.29 (1.03-1.62)**
≥24	517 (50.0)	580 (45.9)	241 (53.0)	195 (47.1)	**0.003**	**1.42 (1.13-1.79)**
Smoking status						
Never	635 (61.5)	845 (66.9)	287 (63.1)	270 65.2)	**<0.001**	**1.44 (1.18-1.76)**
Ever	398 (38.5)	418 (33.1)	168 (36.9)	144 (34.8)	0.1	1.26 (0.96-1.66)
Drinking status						
Never	751 (72.7)	964 (76.3)	341 (74.9)	321 (77.5)	**0.001**	**1.36 (1.13-1.63)**
Ever	282 (27.3)	299 (23.7)	114 (25.1)	93 (22.5)	0.105	1.33 (0.94-1.87)
Family						
No	960 (92.9)	1156 (91.5)	420 (92.3)	379 (91.5)	**0.001**	**1.34 (1.13-1.58)**
Yes	73 (7.1)	107 (8.5)	35 (7.7)	35 (8.5)	0.114	1.65 (0.89-3.08)
Hypertension						
No	638 (61.8)	949 (75.1)	269 (59.1)	310 (74.9)	**0.008**	**1.30 (1.07-1.58)**
Yes	395 (38.2)	314 (24.9)	186 (40.9)	104 (25.1)	**0.008**	**1.47 (1.10-1.97)**
Diabetes						
No	877 (84.9)	1185 (93.8)	418 (91.9)	387 (93.5)	**<0.001**	**1.42 (1.20-1.67)**
Yes	156 (15.1)	78 (6.2)	37 (8.1)	27 (6.5)	0.324	0.74 (0.40-1.35)

* Two-sided χ2 test for number of risk alleles in cases and controls; 95% CI: 95% confidence. Adjusted for age, pack-years of smoking, drinking status, and family history of cancer in logistic regression model.

### Association between CCND1 rs1944129 polymorphism and clinicopathological characteristics of RCC patients

We then investigated the association of the CCND1 rs1944129 polymorphism and clinicopathological characteristics of RCC patients. As shown in Table 6, in patients with stage I, stage II and stage III and moderately (grade I and II) or poorly differentiated (grade III and IV) nuclear grade, no significant difference was observed. However, compared with individuals carrying AG+GG, the rs1944129 AA was significantly more frequent in patients with clinical stage IV (adjusted OR = 52.14; 95%CI = 51.22 - 3.75, *P* = 0.029).

**Table 6:** The association of CCND1 rs1944129 polymorphism and clinicopathologic characteristics of ccRCC patients

Variables	Risk allele				P*	Adjusted OR (95% CI)*
	AA		AG+GG			
	N	%	N	%		
Clinical stage	824		664			
I	520	63.1	426	64.2		1.00 (reference)
II	168	20.4	135	20.3	0.113	0.77 (0.56-1.06)
III	59	7.2	56	8.4	0.611	0.89 (0.56-1.40)
IV	77	9.3	47	7.1	**0.029**	**0.56 (0.33-0.94)**
Grade						
I	175	21.20	144	21.7		1.00 (reference)
II	409	49.60	335	50.5	0.639	0.96 (0.81-1.14)
III	178	21.60	144	21.7	0.670	0.92 (0.62-1.36)
IV	62	7.50	41	6.2	0.818	0.91 (0.42-1.99)

* Two-sided χ2 test for number of alleles in cases and controls; 95% CI: 95% confidence interval. Adjusted for age, BMI, gender, smoking status, drinking status and history of hypertension and diabetes in logistic regression model.

## DISCUSSION

In the present study, we evaluated the associations between CCND1 polymorphisms and the susceptibility and clinicopathological development of RCC in a Chinese population. Our results suggested that genotype variation of rs1944129 polymorphism had a significantly increased risk for RCC, especially in the patients with GG or AG+GG genotypes. Similarly, we also found that individuals with rs7177 genotype variation was associated with increased RCC risk, mainly in those with CC or AC+CC genotypes. However, we could not find any significant difference in the rs9344 and rs678653 genotype frequency between the RCC patients and controls. In addition, because both rs1944129 and rs7177 genotypes had statistically significantly increased risk of RCC, we evaluated rs1944129 and rs7177 together and found that individuals carrying the two or three risk alleles had a significantly increased RCC risk. As far as we know, this study was the first to evaluate the role of the CCND1 polymorphisms in the aetiology of RCC.

As a critical regulatory protein of 295 amino acids, CCND1 gene consists of five exons and four introns and nencodes cyclin D, which affects the transcription of genes and promotes the transition from G1 to S phase of the cell cycle during cell differentiation [19, 20]. It has been demonstrated that cell cycle regulation appears to play a vital role in cell proliferation, differentiation and apoptosis in the evolution and development of several types of cancer, where it can influence the inhibition of mitochondrial metabolism, regulation of transcription factor signaling via a DNA-bound form, the induction of chromosomal instability and so on [21]. Single nucleotide polymorphisms (SNPs) were reported to change the structure of the genome and influence the protein expression and function, contributing to abnormal cell proliferation and an increased risk of cancer [22]. Of the SNPs in CCND1, the mutation is the common and the some allele changes do lead to an alternatively spliced transcript of CCND1, which facilitates the passage of the variant cell through the G1-S checkpoint and rapid proliferation, ultimately resulting in cancer development [23]. Overexpression or disordered regulation of CCND1 is predominantly correlated with early cancer onset, shorter cancer patient survival and increased metastases, and disrupts normal cell cycle process [24–26].

During the past few years, it has been previously reported that high levels of activity of CCND1 polymorphisms could affect the development and progression of certain cancers of humans, such as esophageal squamous cell carcinoma [27], lung cancer [28], breast cancer [29], pancreatic cancer [30] and other cancer types in different ethnicities. For example, Sabir et al. suggested that the CCND1 G/A rs9344 polymorphism was associated with the early onset of head andneck cancer and might contribute to head andneck cancer susceptibility in a Pakistani population [31]. In addition, the A allele of CCND1 rs9344 polymorphism might serve as a risky marker in early detection and prediction for CRC in Taiwan by Huang et al [32]. Nevertheless, studies on the association between genetic variants in CCND1 and susceptibility of RCC were insufficient. With a growing interest in the association between the CCND1 polymorphisms and RCC risk, several studies have tested the hypothesis that the CCND1 polymorphisms were associated with RCC risk [33–35], but the findings remained inconsistent. Therefore, the present study was performed to investigate whether these polymorphisms could influence the susceptibility of RCC.

In our study, we genotyped four polymorphisms in CCND1 to explore the association between CCND1 genetic variants and RCC susceptibility in Chinese population. The CCND1 rs1944129 and rs7177 significantly differed between RCC patients and control participants, indicating that the risk of RCC was increased in participants with the GG or AG+GG genotypes of rs1944129 and CC or AC+CC genotypes of rs7177. The further analysis of the combining risk alleles showed that the group of patients with 2-4 risk alleles was more susceptible to RCC compared with those with 0-1 risk alleles. Environmental and epidemiological factor also had certain effects on the risk of RCC according to our stratification analyses in combination genotypes of CCND1 rs1944129 and rs7177. Age, sex, BMI, smoking status, drinking status, the history of diabetes and hypertension were all related with the RCC susceptibility, implying that the interaction of the environment, hereditary background and genetic variants might be a complex system contributed to the occurrence of RCC [36, 37]. Interestingly, stratification analyses of the association between combination genotypes of CCND1 rs1944129 and rs7177 and the risk of RCC revealed a little different in smokers status, drinking status, history of hypertension or diabetes compared with that of risk alleles. In addition, compared with individuals carrying AG+GG, the rs1944129 AA was significantly more frequent in patients with clinical stage IV.

In reviewing the results of this study, several limitations should be taken into consideration in our present study, some of which cannot be overcame. First, our study was a retrospective hospital-based case-control design, we could not completely rule out the possibility of selection bias for subjects who might have been associated with a particular genotype. Second, the lack of detailed survival data from all participants limited our ability to explore the association between these SNPs in CCND1 and prognosis and survival of RCC. Third, the sample size was relatively small, which reduced the statistical power of combined analysis and stratification, particularly for gene-environment interaction analyses. What's more, our findings needed to be further validated by other high-quality studies with a more comprehensive design in subsequent studies.

## MATERIALS AND METHODS

### Ethics statement

The study was approved by the Institutional Review Board of the Nanjing Medical University, Nanjing, China. At recruitment, a written informed consent was obtained from all participants involved in this study.

### Study population

The present ongoing case-control study of RCC, including 1,488 patients with RCC and a group of 1,677 cancer-free controls was conducted in the Department of Urology, the First Affiliated Hospital of Nanjing Medical University, Nanjing, China, starting in September 2003. In brief, all subjects were genetically unrelated ethnic Han Chinese recruited coming from different families and had no blood relationship. All of the incident RCC patients were newly diagnosed with histopathologically confirmed by two pathologists independently and were identified by reviewing the medical records to ensure no prior history of other cancers or metastasized cancer for other or unknown origins or previously subjected to chemotherapy or radiotherapy, and were consecutively recruited without restriction of age and sex. The cancer-free controls who consisted of randomly-selected volunteers, were seeking health care in the outpatient departments at the hospital matched to the cases’ sex and age (65 years) on frequency. In the present study, nonsmokers were defined as those who smoked less one cigarette per day and less one year over their lifetime and all others were considered as smokers. In addition, drinkers were those who drank at least three times per week for a period lasting more than six months and the rest were defined as never drinkers. Family history of cancer was considered as any occurrence of cancer in first-degree relatives (parents, siblings, or children). The tumor stage was determined using the international tumor-node-metastasis (TNM) classification system and graded according to World Health Organization criteria. Based on the American Joint Committee on Cancer (AJCC), the disease stage was divided into localized group (stage I and stage II) and advanced group (stage III and stage IV) by the assessment of the Fuhrman scale. After signing the agreement, about 5 ml venous blood samples for genomic DNA extraction were obtained from each subject.

### SNP selection

According to HapMap data (http://hapmap.ncbi.nlm.nih.gov/) and PubMed data (http://www.ncbi.nlm.nih.gov/projects/SNP/), some potentially functional tSNPs in CCND1 gene were choosed. Minor allele frequency (MAF) of each gene polymorphism was more than 5% in the Han Chinese population. When some of the SNPs were in complete linkage disequilibrium (r^2^ = 1), only one SNP was selected for genotyping. Finally, we included four SNPs in CCND1 (rs1944129, rs7177, rs9344 and rs678653), which were recently found to be significantly associated with some malignancies in the Chinese population [31, 32, 38].

### DNA extraction and polymorphism genotyping

Total genomic DNA was seperated and purified from the peripheral blood lymphocytes by proteinase K digestion and phenol-chloroform extraction, according to the manufacturer's directions (GoldMag Co. Ltd. Xian, China). The genotyping of CCND1 polymorphisms was performed by pre-designed TaqMan SNP Genotyping Assays (Applied Biosystems, Foster City, CA, USA). The sequence of primers and probes for the SNP were available on request. Amplification was executed under the following melting steps: 2 min at 50°C, 10 min at 95°C, followed by 45 cycles of 95°C for 15 sec and 1 min at 60°C. According to the manufacturer's instructions, amplifications and analysis were adopted by the genotyping assay in the 384-well ABI 7900HT Real-Time PCR System (Applied Biosystems) and the SDS 2.4 software were used to automatically collect and analyze the data for subsequently allelic discrimination in a blind manner. In order to ensure the accuracy of genotyping, positive controls by sequencing and negative controls without DNA were used. Furthermore, four negative controls were included in each 384-well plate for quality control and samples making up more than 5% of the cases and controls were randomly selected for repeated genotyping for confirmation with a reproducibility of 100%.

### Statistical analysis

Student's t-test was performed to test the differences in continuous variables such as age and BMI. Pearson's chi-square test was performed to analyze the differences in categorical variables such as gender, smoking status, drinking status, and in the frequency distribution of CCND1 polymorphisms alleles and genotypes between the cases and controls. The allele frequencies of the CCND1 polymorphisms in the controls were tested against departure from Hardy-Weinberg equilibrium (HWE) by the goodness-of-fit Chi-square test. The adjusted odds ratios (ORs) and 95% confidence intervals (CIs) from unconditional univariate and multivariate logistic regression analysis with the adjustment for possible confounders were used to evaluate the associations between the CCND1 polymorphisms and the risk of RCC. *P* value was deemed as statistically significant when less than 0.05. All the statistical analyses were carried out with the SPSS version 22.0 (SPSS Inc., Chicago, IL, USA) with two-sided P values.

## CONCLUSION

Overall, we investigated an association between CCND1 polymorphisms and susceptibility, clinical characteristics of RCC patients in a large sample population. Our case-control study indicated that CCND1 rs1944129 and rs7177 were the genetic susceptibility factors for the pathogenesis of RCC in Chinese population, and the combination of risk alleles were significantly associated with the elevated risk of RCC. Although the associations appeared to be statistically significant in our population, these findings need to be further validated by other large independent population-base studies with a more comprehensive design and additional available data to investigate the specific function of CCND1 polymorphisms in the development of RCC.
